# A sensorized modular training platform to reduce vascular damage in endovascular surgery

**DOI:** 10.1007/s11548-023-02935-w

**Published:** 2023-05-17

**Authors:** Nikola Fischer, Christian Marzi, Katrin Meisenbacher, Anna Kisilenko, Tornike Davitashvili, Martin Wagner, Franziska Mathis-Ullrich

**Affiliations:** 1 Health Robotics and Automation, Karlsruhe Institute of Technology, Institute for Anthropomatics and Robotics, 76131 Karlsruhe, Germany; 2 Department of Vascular and Endovascular Surgery, Heidelberg University Hospital, Heidelberg, Germany; 3 Department for General, Visceral and Transplantation Surgery, Heidelberg University Hospital, 69120 Heidelberg, Germany; 4 Center for the Tactile Internet with Human in the Loop (CeTI), Technical University Dresden, 01062 Dresden, Germany; 5 Department of Artificial Intelligence in Biomedical Engineering (AIBE), Friedrich-Alexander-University Erlangen-Nürnberg, 91052 Erlangen, Germany

**Keywords:** Sensorized phantom, Impact force sensing, Piezoresistive sensing, Endovascular training

## Abstract

**Purpose:**

Endovascular interventions require intense practice to develop sufficient dexterity in catheter handling within the human body. Therefore, we present a modular training platform, featuring 3D-printed vessel phantoms with patient-specific anatomy and integrated piezoresistive impact force sensing of instrument interaction at clinically relevant locations for feedback-based skill training to detect and reduce damage to the delicate vascular wall.

**Methods:**

The platform was fabricated and then evaluated in a user study by medical ($n=10$) and non-medical ($n=10$) users. The users had to navigate a set of guidewire and catheter through a parkour of 3 modules including an aneurismatic abdominal aorta, while impact force and completion time were recorded. Eventually, a questionnaire was conducted.

**Results:**

The platform allowed to perform more than 100 runs in which it proved capable to distinguish between users of different experience levels. Medical experts in the fields of vascular and visceral surgery had a strong performance assessment on the platform. It could be shown, that medical students could improve runtime and impact over 5 runs. The platform was well received and rated as promising for medical education despite the experience of higher friction compared to real human vessels.

**Conclusion:**

We investigated an authentic patient-specific training platform with integrated sensor-based feedback functionality for individual skill training in endovascular surgery. The presented method for phantom manufacturing is easily applicable to arbitrary patient-individual imaging data. Further work shall address the implementation of smaller vessel branches, as well as real-time feedback and camera imaging for further improved training experience.

**Supplementary Information:**

The online version contains supplementary material available at 10.1007/s11548-023-02935-w.

## Introduction

Endovascular interventions aim for minimally invasive diagnostics and therapy, e.g. on patients with aneurysms, arterial occlusive disease or strokes. Such procedures require skillful insertion and steering of different instruments (i.e. guidewires, catheters) through the vascular system of the patient. The objective is to reach the actual site of operation where stent placement, coil placement or thrombus removal take place. Challenges include the restricted visual and haptic feedback regarding the delicate manipulation of the instruments within the vascular system. Here, exerted force can easily damage the delicate vascular wall (endothelium) that may lead to dissection and consecutive dangerous perfusion impairment. While modern visual imaging techniques such as intraoperative computed tomography angiography (CTA) with contrast agent improve the visualitation, instrument handling remains a difficult cognitive and dexterous task, requiring years of training.

Clearly, teaching and training in a real operation scenario and on a living patient is indispensable. However, such trainee positions and training time are limited as well as patient pathologies and anatomies are limited to cases appearing in the hospital. Furthermore, beginners may introduce a higher risk of complications despite being supervised by an experienced mentor. Realistic procedure simulations can provide effective training outside of the operating room and lead to efficiency improvement [1], as for example Kendrick et al. demonstrated for a thoracic endovascular aortic repair procedure [2].

A major complication includes damage to the vessel walls [3]. Thus, high impact forces from the instrument to the inner vessel walls must be avoided and training simulations must consider this risk factor. Long before entering an operation room, trainees need to familiarize with instruments and techniques in a safe and radiation-free environment, ideally on a model featuring realistic anatomical patient-specific structures and instrument interaction.

Modern training platforms and simulators not only provide realistic haptics but also a feedback modality to relieve teachers from attending repetitive, time consuming training sessions. Furthermore, such platforms provide qualitative and quantitative data to obtain comparative results and progress of training.

Aggarwal et al. compare the training simulators *Angio Mentor* (Simbionix USA Corp, USA) and *VIST* (Mentics AB, Sweden), which measure collision and frictional forces during the intervention with real-time feedback [4]. Furthermore, the authors considered virtual reality (VR) enhanced systems as the *CathLab VR* (CAE Healthcare, Canada), which were demonstrated beneficial to training progress [5]. Another commercially available simulation platform is the *CATHiS* (CATHI GmbH, Germany), a highly compact and mobile system with strong focus on realistic simulation of visual imaging, force feedback and patient complications. In contrast to this black-box simulator, the *Endovascular Evaluator EVE* (BR Biomedical (P) Ltd., India) comes with silicone vessel models that mimic the arteries’ elasticity and friction coefficients.

However, high costs for purchase and maintenance [4, 6] of such systems exclude many young physicians from such a learning opportunity that potentially increase patients’ safety and reduce teachers’ obligations in this crucial part of training in endovascular surgery.

Besides commercially available solutions, several research groups investigate 3D printing of vessel phantoms and proof their benefit to facilitate surgical planning in endovascular interventions and other fields of application prior to the operation [7, 8]. Kaschwich et al. propose a simulator consisting of 3D-printed patient-specific phantom parts that are ultrasound capable for enhanced simulation scenarios [9]. To improve training, Payne et al. present a leader-follower system to insert and navigate a catheter, providing an intuitive controlling device, which can create a haptic feedback based on force gauges at the catheter’s distal tip [10], rendering essential modification of the instrument. Chi et al. demonstrate a platform for online skill assessment featuring haptic guidance to learn by expert demonstration that also includes force feedback [11]. However, the handheld device interfacing the user to the catheter may lead to an alienation instead of familiarization with a real instrument. Furthermore, Rafii-Tari et al. developed a simulation platform that measures impact forces by placing the entire phantom on a force-torque sensor and tracking catheter motion with rotary encoders [12]. While bearing the system on a sensor makes the platform more difficult to use, the encoders create an additional source of externally induced friction and resistance and thus influence the training unintentionally.

**Fig. 1:**
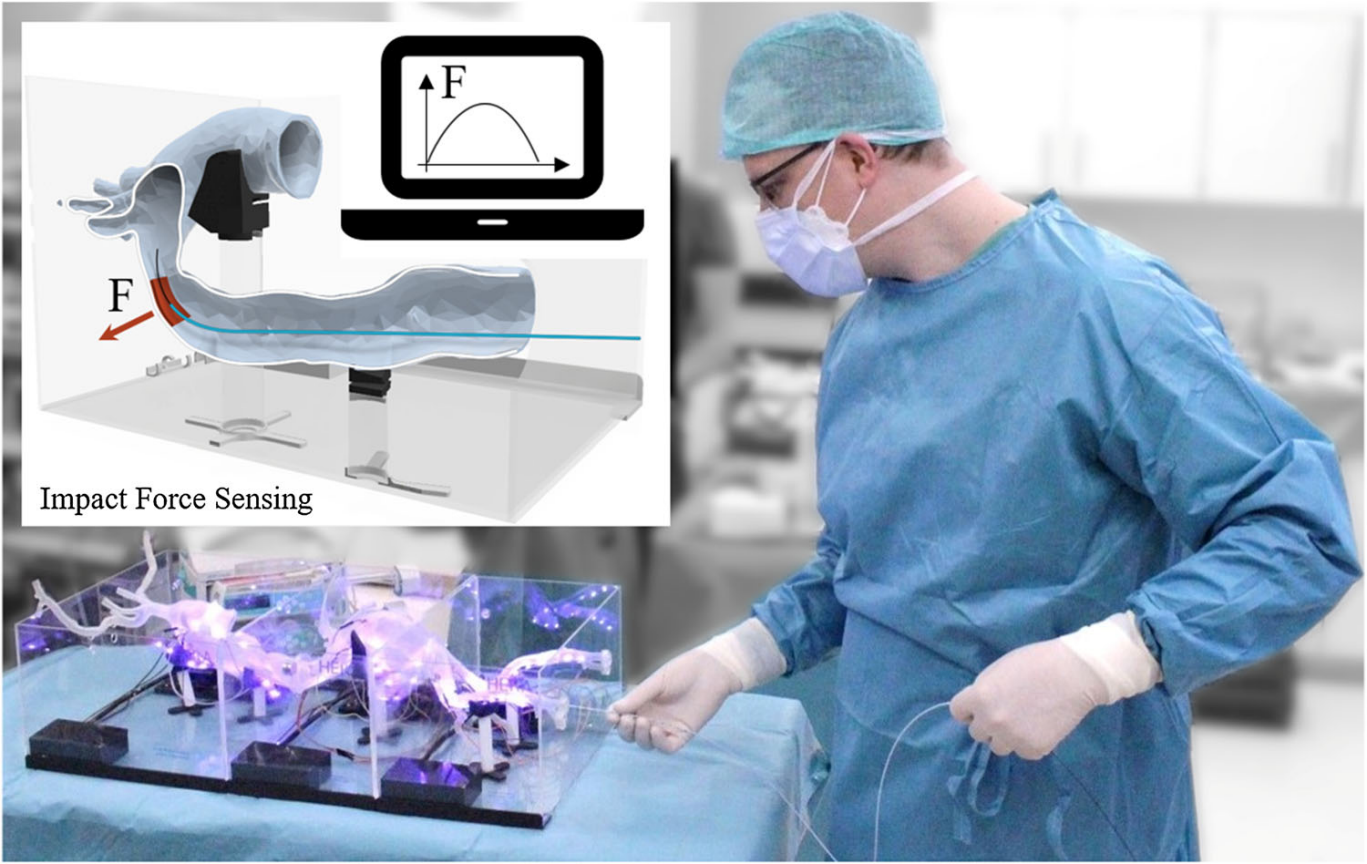
Schematic of the impact force sensing training platform (top) and experimental evaluation of the modular demonstrator at Heidelberg University Hospital

The aforementioned systems are usually preset for one specific anatomy. However, training could strongly benefit from a modular system that can represent different anatomical parts for specific training episodes. Furthermore, the possibility to easily include patient-individual pathologies in the training phantoms would enable specific training and planning prior to an intervention. Most simulation platforms that include some force sensing modality address haptic feedback but at the cost of additional modifications of the instruments and control devices that omit hands-on experience with the instruments. Therefore, we propose an authentic training platform, consisting of multiple independent modules with 3D-printed vessel phantom sections and integrated impact sensing based on piezoresistive sensors for training of endovascular interventions providing feedback to the trainees (Fig. 1). The platform concept allows for customization by creating phantom parts based on real patient CTA-data and mobile usage of single parts or the entire unit to skill labs or lecture halls.

**Fig. 2:**
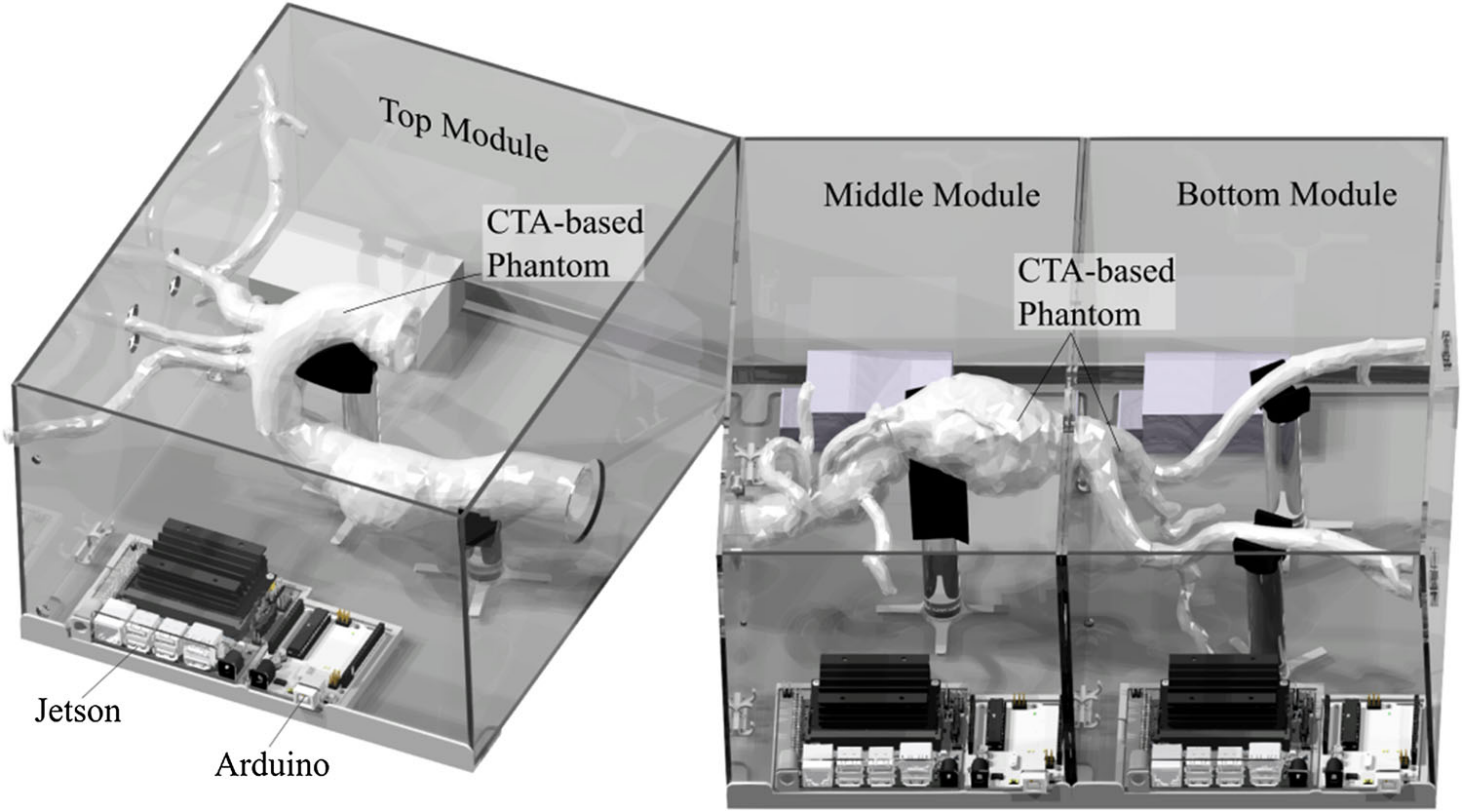
Render of the 3 modules of the training platform in a computer-aided design (CAD) model. Each module comprises the respective section of the individualized vessel phantom as well as hardware for sensing and processing

## Materials and methods

### Platform design

The training platform is shown in Fig. 2 and covers the complete aorta without organs (heart, lungs, liver, kidneys) from the supraaortic arch vessels downstream to the *A. poplitea*. It is subdivided into three modules of relevance, which can be operated individually or combined to create desired training scenarios. Following the classification according to Fillinger et al. [13], the platform includes aortic zones 0–5 (top module), 6–9 (middle module) and 10–11 (bottom module). Each module features three piezoresistive sensors to measure impact forces on the vessel walls. Instruments can be inserted via the right *A. femoralis*.

**Fig. 3:**
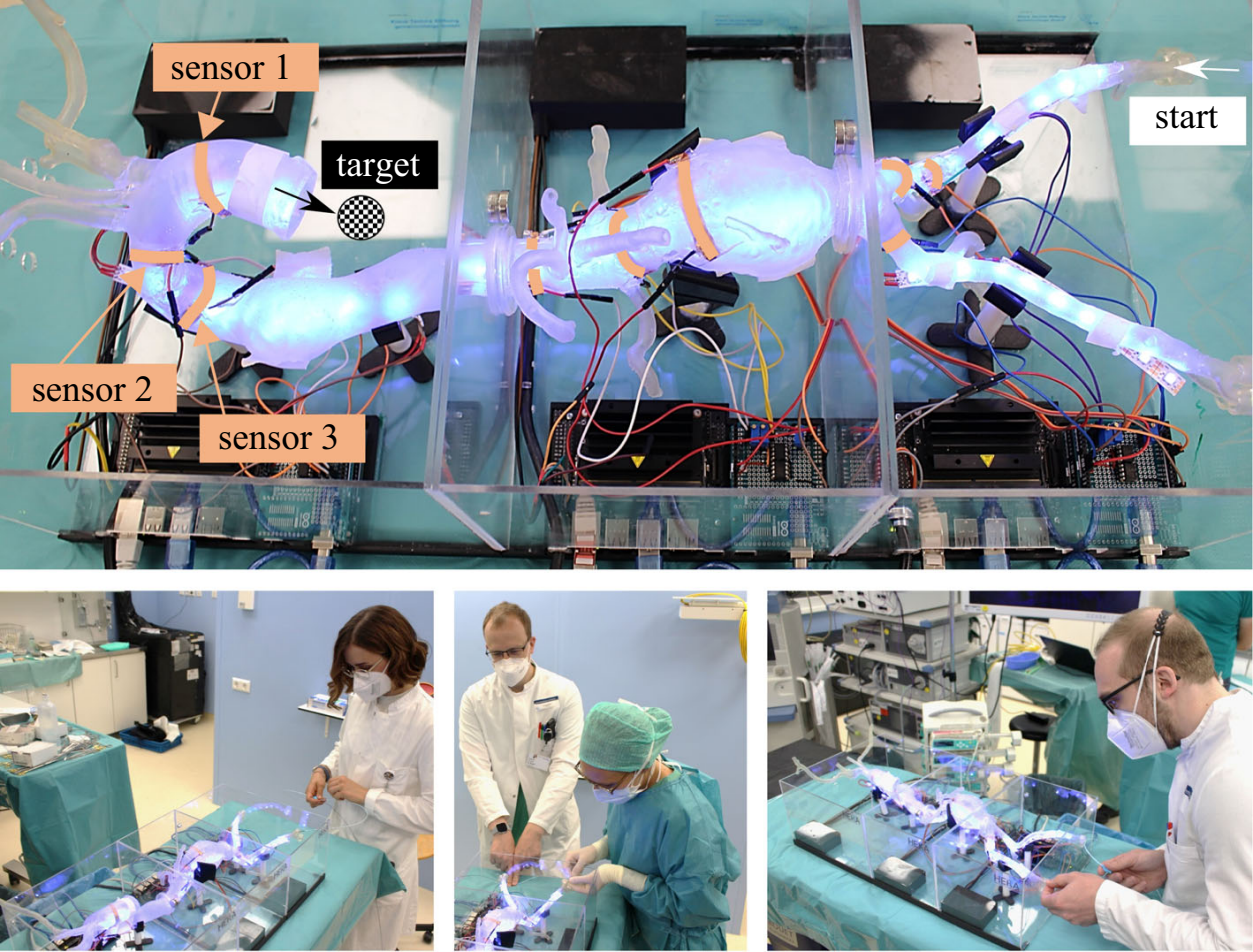
The modular sensorized training platform as used for the user study. Indicated start and target positions between a total of three module boxes, as well as the three sensor locations per box (orange). Bottom: Medical experts during the user study

### Phantom fabrication

The vessel phantom is based on segmentation of anonymized CTA-data of a patient with an aneurysm of the abdominal aorta. The processing of the CTA-data and fabrication method allows for any patient data to be turned into an authentic and customized training phantom. From the CTA-data the aorta is segmented using The Medical Imaging Interaction Toolkit (MITK) as a single connected volume, exported as single raw shape and stored as.stl-file. Then, Blender is used to derive a model of the vessel wall with 1.5 mm thickness. Subsequently, this model is cut along the border of the boxes to obtain the respective models for each module box. These subsegments are then 3D-printed using a stereolithography printer (Form3, Formlabs GmbH, Germany) with a special resin that remains elastic after curing (Elastic 50A, Formlabs GmbH, Germany) to provide realistic haptics. The subsegments can be joined after curing, by manually applying liquid resin and curing it with a UV-flashlight. Thus, larger and more complex vessels can be fabricated. The cured resin is translucent and allows the user to observe instruments within the phantom. Aiming on recreation of low friction between instrument and vessel wall, a preliminary experiment was conducted to select a low friction coating for the inner vessel walls. A catheter was extracted from a simplified phantom with a motorized setup while measuring forces. Out of three investigated coatings, a mineral spray oil (Komet, Fessmann & Hecker GmbH & Co.KG, Germany) provided minimal wall friction and was used to coat the inner vessel walls of the phantom. Due to the hydrophilic coating of many commercial catheters also a water based coating was investigated. However, it was ruled out due to the risk of harming sensor electronics while not providing measurable benefits on friction. For the coating process, the phantom received a base coating by covering it in lubricant and letting excess oil drip off. During preparation of the users studies the coating was refreshed by applying small amounts to not form droplets or letting oil accumulate at the phantom’s bottom.

### Impact force sensing

The phantom vessel walls must remain elastic but at the same time detect impact forces that would be harmful to real vessel tissue. Therefore, flexible compliant sensors are required for detection of impacts of the instrument tip. In addition, the buckling instrument might push against the vessels walls, e.g. when probing. Sensors made from piezoresistive polymers are well suited for this usecase as presented in [14]. These can be fabricated in almost arbitrary shapes, at low thickness of $<~{400}~{\upmu \hbox {m}}$ and were initially demonstrated by Fischer et al. to measure impact forces of up to 1 N with 0.1 N resolution.

Here, such sensors are fabricated and placed at the circumference of the vessel phantom at selected locations on the inner vessel walls (Fig. 3). In discussion between technical and medical experts, these locations were chosen according to high-impact zones for a typical catheter trajectory. For example, we chose regions, where the catheter must take sharp turns to reach smaller vessels, the aortic bifurcation or at the aortic arch. The sensors must be attached on the inner vessel walls so that the sensor surface gets in direct contact to the instrument. Together with the elastic vessel wall, the sensor strip deforms at impact and provides an estimation of the impact force.

### Data processing

Each module box must show full functionality when operated stand-alone and in any anatomically meaningful combination with adjoining module boxes. While the box walls provide customized cuffs to interconnect them mechanically, also the sensor data acquisition and data processing must contribute to the modular concept. Therefore, each module box is equipped with a mini computer (NVIDIA Jetson Nano, Nvidia Corporation, USA) with Ubuntu 20.04 as operation system and Robot Operating System (ROS) noetic. Thus, each module box can be set up to acquire, process, distribute, and monitor its own sensor data as well as the data of all other adjoining module boxes in the network. Each module box also features an Arduino Uno (ATmega328-Microcontroller) to control and power the sensors and to feed the computed impact force data via serial connection to the minicomputer.

## Experimental evaluation

To evaluate the training platform, a user study was conducted at Heidelberg University Hospital. We investigated whether the platform allows for discrimination between different levels of experience and expertise, as well as if young physicians could improve their skills in catheter and guidewire handling by training with it.

For the study, board-certified surgeons ($n=2$), surgical residents ($n=3$), medical students ($n=5$), and non-medical users ($n=10$) tested the training platform in five runs each. Their performance was assessed by measuring mean runtime *t*, mean impact force *F*, and calculating an impact-runtime score $\chi$. The latter was defined as the unweighted product $\chi = t \cdot |F |$ in order to obtain a user-specific ordinal value to reflect performance including both metrics.

To analyze the influence of **expertise** on the performance on the training platform, the users are subdivided into groups of medical experts, medical students, and non-medical users. 5 of the 10 medical users already had experience with the instruments on any phantom (2 users) and on a patient (3 users). 3 of the non-medical users were already familiar with the instruments and especially with the actual training platform. Then, the users are regrouped according to their prior **experience** with catheters and guidewires on phantoms and patients, respectively. Eventually, an analysis of variance (ANOVA) is conducted (MATLAB R2022a, Mathworks Inc., USA) to determine statistical significance in the differences of expertise and experience on performance.

### Experimental setup

Figure 3 shows the experimental setup with a total of three module boxes in operation. Each box was equipped with three force sensors (orange). To increase the visibility of the instruments for the users, the phantom was illuminated with blue light from the bottom side in addition to the ambiance illumination of the experimental operation room. Furthermore, the defined starting point (femoral) and the end point (aortic arch) are illustrated. On the path in between, the instruments pass through the module boxes from right to left, which corresponds to a realistic intervention. The utilized instruments were a guidewire (RADIFOCUS: M, 0.035” Flex L 3 cm, 180 cm stiff type *angled*, Terumo Interventional Systems, Belgium) and a matching catheter (Accu-Vu Pigtail, 5F 1.8 mm$\times$100 cm, AngioDynamics, USA).

**Fig. 4:**
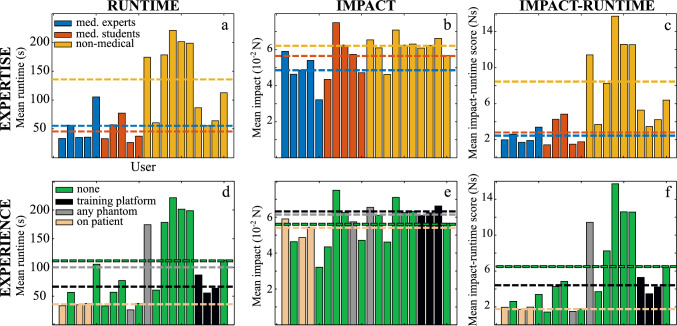
Mean values over 5 runs and all sensors for runtime *t* (**a**, **d**), impact force *F* (**b**, **e**), and impact-runtime score $\chi$ (**c**, **f**) for each user. Analysis of user groups based on their expertise (top row) and experience (bottom row) with the mean values for each group (dashed line)

**Fig. 5:**
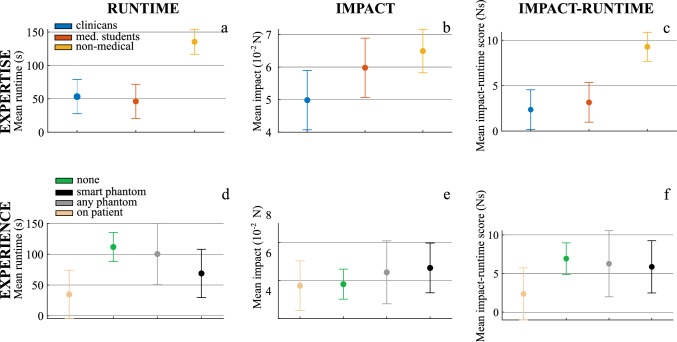
Analysis of variance for the user study. Displayed are estimated means and comparison intervals for runtime *t* (**a**, **d**), impact force *F* (**b**, **e**), and impact-runtime score $\chi$ (**c**, **f**), for groups of expertise (top) and experience (bottom)

### Experimental procedure

Before the actual experiments, the 20 users were individually instructed about the experimental procedure and handling of the guidewire and catheter. The movement possibilities (degrees of freedom) and configurations were explained. In addition, reference was made to the objective of completing the parkour in the shortest possible time and with the lowest possible impact forces between instruments and phantom vessel walls. Prior to each run, the catheter was placed at the starting position (start) in the phantom. The experiment was started with an acoustic signal. The goal was to move the combination of guidewire and catheter to the exit of the aortic arch (target) and then remove the instruments completely from the phantom. The removal process was integrated into the procedure because impacts on the vessel wall causing eventual damage could also occur during removal. If an experimental run was not completed within 300 s, users were instructed to remove the instruments from the phantom immediately, regardless how far they had progressed. Preliminary tests revealed that runtimes were usually below 150 s. With a safety margin of twice that duration we aimed to capture the majority of probands’ interaction with the phantom. For each run, the impact force at each sensor and duration were measured. The runs followed each other without breaks. After completion of all runs, users completed a questionnaire in which they provided general information about their previous experience and suggestions for improvement (see Suppl. 1). The board-certified surgeons, who had been familiar with interventional/endovascular surgery in their clinical routine, were asked additionally how realistic they considered the training platform and whether they would recommend it for surgical training.

**Fig. 6:**
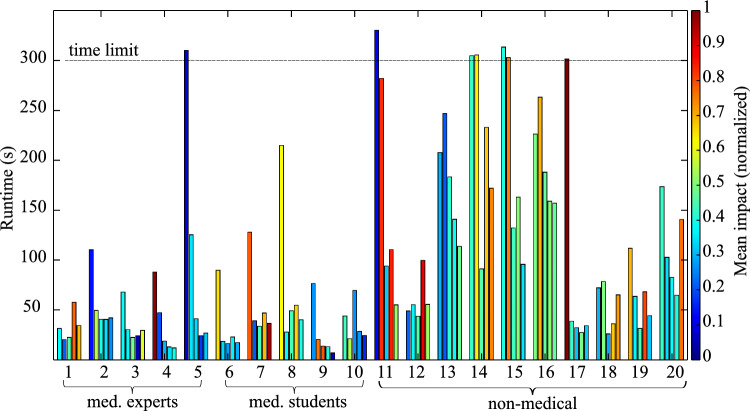
Runtime of all medical users (1–10) and non-medical users (11–20) for all runs. Color-code indicates mean impact force *F* over all sensors, normalized for 0.2 N

## Results and discussion

### User study: experience and expertise

Figure 4 shows the results of the evaluation with users grouped according to their **expertise** (top row) and their **experience** (bottom row), respectively.

When grouped following their **expertise**, medical users require on average approximately one third of the runtime of the non-medical group with a mean of ${46.2}~{\hbox {s}}\pm {20.8}~{\hbox {s}}$ (students) resp. ${53.3}~{\hbox {s}}\pm {30.8}~{\hbox {s}}$ (experts) compared to ${135.4}~{\hbox {s}}\pm {65.9}~{\hbox {s}}$ (non-medical), as seen in Fig. 4a. Thereby, the medical students require a comparable duration as the medical experts. However, the latter achieve the lowest mean impact force *F* with ${0.048}~{\hbox {N}}\pm {0.010}~{\hbox {N}}$ in contrast to ${0.057}~{\hbox {N}}\pm {0.013}~{\hbox {N}}$ (students) and ${0.062}~{\hbox {N}}\pm {0.007}~{\hbox {N}}$ (non-medical), as seen in Fig. 4b. Consequently, the impact-runtime score $\chi$ is lowest with the medical experts (${2.33}~{\hbox {N s}}\pm {0.69}~{\hbox {N s}}$), closely followed by the medical students (${2.77}~{\hbox {N s}}\pm {1.65}~{\hbox {N s}}$). Non-medical users obtain an impact-runtime score $\chi$ of ${8.36}~{\hbox {N s}}\pm {4.41}~{\hbox {N s}}$ on average (Fig. 4c). The group comparison based on the analysis of variance (*cf.* Fig. 5) reveals runtime ($p\le 7.6\cdot 10^{-5}$) and impact-runtime ($p\le 9.4\cdot 10^{-5}$) as valid factors for significant differentiation of medical and non-medical users. In contrast, force impact can only be used to distinguish medical users from the non-medical group. Thus, runtime seems to be the more meaningfully metric for identification of experience as well as expertise and as such more valuable as training feedback. However, the impact metric could become more relevant, when researching risk of vessel puncture in future work.

When grouped following their **experience**, the medical experts with previous experience on patients require the least mean runtime *t* of ${34.7}~{\hbox {s}}\pm {1.3}~{\hbox {s}}$ (Fig. 4d) and apply the lowest mean impact force *F* of ${0.05}~{\hbox {N}}\pm {0.005}~{\hbox {N}}$ (Fig. 4e) in contrast to the comparison groups with only limited or no experience on phantoms. The data also show that the users with previous experience on this particular training platform are not faster and work with greater forces than the experienced medical professionals. The advantage of real experience seems thus represented well by the impact-runtime score $\chi$ of our platform. Users without previous experience performed similarly well as those who had already gained experience on other phantoms since both groups achieved an impact-runtime score $\chi$ of ${6.46}~{\hbox {N s}}$ with $\pm {4.77}~{\hbox {N s}}$ (none) and $\pm {7.01}~{\hbox {N s}}$ (any phantom) respectively (Fig. 4f). This indicates that previous experience with other phantoms is not directly transferable to our training platform. However, the low number of users in this group make statistical comparison difficult, as also indicated by the high standard deviation in this group with only two group members.

Statistically, the combined score $\chi$ cannot be used for differentiation of users with experience on a phantom or the training platform ($p\le 0.67$). Significant difference can only be shown for user with experience on patient, compared to no experience ($p=0.0095$), as seen in Fig. 5(d+f). The low impact-runtime score $\chi$ of the medical experts with experience on patients confirms the superiority of this expert group over all comparison groups. This correspondence is in agreement with the expected real distribution of experience and skills.

The grouping in this study by experience provides some further insights, but must be critically examined due to the potential in-homogeneity inside of the medical and non-medical group mentioned in “Experimental evaluation” section. Furthermore, from the medical group, there was only one person from the specialty of (endo-)vascular surgery, while most medical experts were general and visceral surgeons. In addition, potentially controversial is the different number of members in the subgroups. Thus, 12 users with no experience were compared to only 2 users with previous experience on other phantoms. Due to the relatively small sample size, the statistical evaluation of the present study may not be representative for a larger population. Yet, it could be shown that the platform allows gathering of metrics which provide differentiation between professional users based on experience and expertise. Thus, these metrics can provide valuable feedback for training and education.

### User study: learning curve

Figure 6 shows the runtime of all users for each of the 5 runs and the mean impact force *F* across all sensors (normalized to 0.2 N) and visualized in color code. This allows examination of each user’s learning curve (i.e., runtime and impact) throughout the experiments indicating the training effect. Most users require longer runtimes initially and become faster towards the end of the experimental series. With the exception of users 1, 12 and 20, each last run was the fastest run. Although starting in the first run with low runtime and impact, performance of user 1 (vascular surgeon, 8 years of professional experience) is first improving (run 2) and then lowering towards the last runs. We can assume them to having already completed most of their learning curve long before this study.

Overall, it can be observed that the runtime varies considerably. For example, the total of terminations of runs due to exceeding maximum runtime of users 5, 11, 14, 15 and 16 strongly differ to a fastest duration of only 7.1 s (user 9). We can conclude that the 7 terminations out of a total of 100 runs confirm the functionality of the training platform and the feasibility of the task proposed in this study. All users were able to conduct at least 3 complete runs successfully without exceeding the time limit of 300 s.

Regarding impact, 11 out of the 20 users showed an increased impact force on the vessel walls after 5 runs, while 8 decreased their impact force level and one user (16) remained almost the same. During the tests, it was observed that the users’ self-confidence seemed to increase after each run. Whereas at the beginning there was still tentative trial and error out of careful consideration for the training platform, it was quickly understood that it could withstand with its robust construction the stresses and that progress could be accelerated with greater effort, especially through dexterous repetitive rapid forward and backward movements. Also, many users quickly found that the task set could be solved without pronounced configuration changes, as there was no need to probe steep vascular branches that would have required advanced skills with the catheter and guidewire. Such more complex tasks could be envisioned for future studies. This would be expected to result in even clearer distinction between experts and non-experts. Four medical (1, 2, 5, and 7) and 6 non-medical (11, 12, 13, 14, 18, and 20) users did not improve impact forces, when comparing their first and the last run. This means that half of the users were unable to reduce the impact force over 5 runs, independent of their background. A closer look at this group reveals that of a total of 5 medical students, 4 were able to improve, whereas among the medical experts, only 1 out of 5 was able to do so. Most medical experts already demonstrated a better performance from the beginning, compared to students and non-medical users which explains the lack of apparent improvement.

In summary and with special regards to the target group of young physicians, all could reduce their runtime and 80% also the force impact from run 1–5. Similar to the results from the analysis of expertise and experience it can be seen, that impact readings do not display the same learning effect as seen for runtime. In this work, it still remains an open question how the sensory data can be translated to risk of vessel rupture. If such a metric is found, future studies with the presented phantom could look into increasing the learning effect with direct visual or auditory risk feedback.

### Evaluation of the user questionnaire

The medical experts considered the advancement of the catheter inside the phantom to be moderately realistic compared to a real patient. On average, the experienced surgeons rated the resulting friction between instrument and phantom higher than in vivo. The experts agreed that retracting the catheter and the associated friction was realistic compared to the real patient. In the free-text statements, it was criticized that the individual modules of the platform were not attached to each other well enough so that the catheter could slide out between the modules during probing. Furthermore, the high friction of the system was criticized in general, as well as the insufficient monitoring of the instrument position within the phantom in particular sections. Interestingly, the sensing electrodes on the inner phantom walls, which create presumably the highest friction, were compared to atherosclerotic plaques, which can also cause increased friction in real patients. Overall, the training platform was well received by both, medical experts and non-medical users. The users consider the platform highly promising and useful for medical education. For further improvement, they recommend a user interface with real-time feedback and quantitative measurement of the learning success.

## Conclusion

A training environment for hands-on training in endovascular surgery was investigated as an evaluation platform with realistic geometry and haptic experience, compatible with endovascular instruments such as catheters and guidewires. The presented method of manufacturing can be applied to create various patient-specific anatomies and pathologies of vessels and organs, featuring customized sensing capabilities throughout the elastic phantom structures. The conducted user study shows in a first attempt, that the platform can enhance conventional radiation-free surgical skill training without endangering patients. The study’s results are limited by the size and in-homogeneity of the user cohort, as well as the fact that even though the manufacturing method is transferrable to other patient’s anatomies, it was only shown with one individual and the combination of all three modules. Impact measurement was not yet analyzed sufficiently to allow for inferring of risk for vessel rupture. Preliminary studies only evaluated the measurement on normal forces without regard for shear forces and friction. Further developments will include the application of the manufacturing on other, even more delicate vessel systems such as in the brain, increasing sensor resolution and respond times towards real-time feedback ability, as well as camera imaging for better training.

## Supplementary Information

Below is the link to the electronic supplementary material.

Supplementary file 1 (pdf 58 KB)
