# Supplementary material for: A sensorized modular training platform to reduce vascular damage in endovascular surgery
Source: Int J Comput Assist Radiol Surg. 2023 May 17;18(9):1687–95. doi: 10.1007/s11548-023-02935-w (PMC10491519; doi:10.1007/s11548-023-02935-w)
Supplement: Supplementary file 1 — (pdf 58 KB) [file 11548_2023_2935_MOESM1_ESM.pdf]

**Supplementary Information (SI)**

**Article title:**

A Sensorized Modular Training Platform to Reduce Vascular Damage in Endovascular Surgery

**Journal name:**

International Journal of Computer Assisted Radiology and Surgery

**Authors:**

Nikola Fischer[1], Christian Marzi[1], Katrin Meisenbacher[2], Anna Kisilenko[3], Tornike Davitashvili[3], Martin Wagner[3,4] and Franziska Mathis-Ullrich[1,5*]

[1]Health Robotics and Automation, Karlsruhe Institute of Technology, Institute for Anthropomatics and Robotics, Karlsruhe, 76131, Germany.
[2]Department of Vascular and Endovascular Surgery, Heidelberg University Hospital.
[3]Department for General, Visceral and Transplantation Surgery, Heidelberg University Hospital, Heidelberg, 69120, Germany.
[4]Center for the Tactile Internet with Human in the loop (CeTI), Technical University Dresden, Dresden, 01062, Germany.
[5]Dep. Artificial Intelligence in Biomedical Engineering (AIBE), Friedrich-Alexander University, Erlangen, 91052, Germany.

*Corresponding author: franziska.ullrich@kit.edu

**Submission date:**

9th January 2023

**Supplement 1 - Content:**

This supplementary information feature a **User Questionnaire** conducted with all users of the user study conducted at Heidelberg University Hospital and Karlsruhe Institute of Technology.

**User Questionnaire**

1. First Name, Name

_______________________________

2. You belong to which group?

- ☐ Medical Expert
- ☐ Medical Student
- ☐ Non-medical User

3. What is your medical specialty?

- ☐ Vascular Surgery
- ☐ General Surgery
- ☐ No specialty yet (med-student)
- ☐ No specialty (non-medical user)

4. Prior to these experiments I have already used a catheter or guidewire on ...

- ☐ ... no phantom and no human vessels.
- ☐ ... this phantom.
- ☐ ... another phantom.
- ☐ ... real human vessels (if checked: please CONTINUE with questions below).

5. ADVANCING the catheter/guidewire appears the SAME as on the real patient.

| | 1 | 2 | 3 | 4 | 5 | |
|---|---|---|---|---|---|---|
| strongly disagree | | | | | | strongly agree |

6. ADVANCING the catheter/guidewire creates friction which is too ... compared to a real patient.

| | 1 | 2 | 3 | 4 | 5 | |
|---|---|---|---|---|---|---|
| low | | | | | | high |

7. PROBING appears the SAME as on the real patient.

| | 1 | 2 | 3 | 4 | 5 | |
|---|---|---|---|---|---|---|
| strongly disagree | | | | | | strongly agree |

8. PROBING creates friction which is too ... compared to a real patient.

| | 1 | 2 | 3 | 4 | 5 | |
|---|---|---|---|---|---|---|
| low | | | | | | high |

9. PULLING OUT the catheter/guidewire appears the SAME as on the real patient.

| | 1 | 2 | 3 | 4 | 5 | |
|---|---|---|---|---|---|---|
| strongly disagree | | | | | | strongly agree |

10. PULLING OUT the catheter/guidewire creates friction which is too ... compared to a real patient.

| | 1 | 2 | 3 | 4 | 5 | |
|---|---|---|---|---|---|---|
| low | | | | | | high |

11. What would make artery probing more realistic in the vessel segment sample?

12. The vessel segment was mounted too ... during testing to appear realistic compared to a real patient.

| | 1 | 2 | 3 | 4 | 5 | |
|---|---|---|---|---|---|---|
| firmly | ○ | ○ | ○ | ○ | ○ | loose |

13. How realistic would you rate the haptic experience with the training platform overall?

| | 1 | 2 | 3 | 4 | 5 | |
|---|---|---|---|---|---|---|
| highly unrealistic | ○ | ○ | ○ | ○ | ○ | highly realistic |

14. How would you rate the visibility of the catheter?

| | 1 | 2 | 3 | 4 | 5 | |
|---|---|---|---|---|---|---|
| low | ○ | ○ | ○ | ○ | ○ | high |

15. How useful would you rate the training platform for medical training?

| | 1 | 2 | 3 | 4 | 5 | |
|---|---|---|---|---|---|---|
| useless | ○ | ○ | ○ | ○ | ○ | useful |

16. Which aspect of the phantom is most unrealistic? What would be needed to be improved the most to serve as a training device?

17. What important Feedback could you give that was not covered by the survey?

18. Further Remarks
